# Effective gene prediction by high resolution frequency estimator based on least-norm solution technique

**DOI:** 10.1186/1687-4153-2014-2

**Published:** 2014-01-04

**Authors:** Manidipa Roy, Soma Barman

**Affiliations:** 1 The Calcutta Technical School, Govt. of West Bengal, 110,S.N.Banerjee Road, Kolkata 700013, India; 2 Institute of Radio Physics & Electronics, University of Calcutta, 92, A.P.C. Road, Kolkata 700 009, India

**Keywords:** Periodogram, Deoxyribonucleic acid, Least-norm solution, Eigenvector, Eigenvalue

## Abstract

Linear algebraic concept of subspace plays a significant role in the recent techniques of spectrum estimation. In this article, the authors have utilized the noise subspace concept for finding hidden periodicities in DNA sequence. With the vast growth of genomic sequences, the demand to identify accurately the protein-coding regions in DNA is increasingly rising. Several techniques of DNA feature extraction which involves various cross fields have come up in the recent past, among which application of digital signal processing tools is of prime importance. It is known that coding segments have a 3-base periodicity, while non-coding regions do not have this unique feature. One of the most important spectrum analysis techniques based on the concept of subspace is the least-norm method. The least-norm estimator developed in this paper shows sharp period-3 peaks in coding regions completely eliminating background noise. Comparison of proposed method with existing sliding discrete Fourier transform (SDFT) method popularly known as modified periodogram method has been drawn on several genes from various organisms and the results show that the proposed method has better as well as an effective approach towards gene prediction. Resolution, quality factor, sensitivity, specificity, miss rate, and wrong rate are used to establish superiority of least-norm gene prediction method over existing method.

## 1 Introduction

It has been observed that the most significant scientific and technological endeavour of the 21st century is mostly related to genomics. Therefore, researchers from various cross fields have concentrated in the field of genomic analysis in order to extract the vast information content hidden in it. Deoxyribonucleic acid (DNA) is the hereditary material present in all living organisms. In eukaryotic organisms, genes (sequences of DNA) consist of exons (coding segments) and introns (non-coding segments). It has been established that genetic information is stored in the particular order of four kinds of nucleotide bases, Adenine (a), Thymine (t), Cytosine (c) and Guanine (g) which comprise the DNA biomolecule along with sugar-phosphate backbone. Exons of a DNA sequence are specified as the most information-bearing part because only the exons take part in protein coding while the introns are spliced off during protein synthesis process. Gene prediction means detecting locations of the protein-coding regions of genes in a long DNA chain. Since DNA encodes information of proteins, various statistical and computational techniques have been studied and explored to extract the information content carried by DNA and distinguish exons from introns.

Genomic information is made up of a finite number of nucleotides in the form of alphabetical characters; hence, it is discrete in nature. As a result, digital signal processing (DSP) techniques can be used as effective tools to analyze DNA in order to capture its periodic characteristics. The main objective of spectrum estimation is determination of power spectrum density of a random process. Power spectral density (PSD) describes how the average power of a signal x[n] is distributed with frequency, where x[n] is a sequence of random variables defined for every integer n. The estimated PSD provides information about the structure of a random process which can be used for refined modeling, prediction, or filtering. Estimation of power spectrum of discretely sampled processes is generally based on procedures employing the fast Fourier transform (FFT). This approach is computationally efficient and produces reasonable results, but in spite of the advantages, it has certain performance limitations. The most important limitation lies in its frequency resolution. Moreover, spectral estimation by the Fourier method generates various harmonics which often lead to false prediction of coding regions. Among the recently introduced techniques, the eigendecomposition-based noise subspace method, known as the least-norm solution is found to be of great interest. In the present paper the authors addressed the problems posed by standard FFT method and proposed a least-norm algorithm based on the concept of subspace frequency estimation for effective and accurate prediction of coding regions in DNA sequence.

Application of DSP methods to find periodicities in DNA sequences has been studied by various researchers [1-4]. It is established that exon regions of DNA molecules exhibit a period-3 property because of the codon structure involved in the translation of nucleotide bases into amino acids [5-7]. Yin and Yau explained the phenomenon of three-base periodicity in the Fourier power spectrum of protein-coding regions resulting from nonuniform distribution of nucleotides in the three codon positions [8]. An improved algorithm for gene finding by period-3 periodicity using the nonlinear tracking differentiator is presented by Yin et al. [9]. Peng et al. discussed about statistical properties of genes in their article [10]. A universal graphical representation method based on S.S.-T. Yau’s technique employing trigonometric functions which denotes the four nucleotide bases to predict coding regions is presented by Jiang et al. [11]. Application of digital filters to extract period-3 components and effectively eliminate background noise present in DNA sequence has given good results [12-14]. Yu et al. have used in their paper probability distributions to study similarity in DNA sequences employing symmetrized Kullback–Leibler convergence [15]. Kwan et al. introduced novel codes for one-sequence numerical representation for spectral analysis and compared them with existing mapping techniques [16]. Roy et al. introduced positional frequency distribution of nucleotides (PFDN), an algorithm for prediction of coding regions [17]. Parametric techniques of gene prediction where autoregressive all-pole models were used for identifying coding and non-coding regions provided better results [18,19]. Yu et al. proposed a novel method to construct moment vectors for DNA sequences using a two-dimensional graphical representation and proved that the two had one-to-one correspondence [20]. In another work, Deng et al. introduced a novel method of characterizing genetic sequence defining genome space with biological distance for subsequent applications in analyzing and annotating genomes [21]. An exclusive survey of various gene prediction techniques is presented by Pradhan et al. [22]. The fundamental theory of principal component analysis is explained by Shlens and its application is discussed by Ubeyli et al. [23,24].

In this article, authors have compared and analyzed power spectral peaks obtained by modified periodogram method with pseudo-spectrum obtained by least-norm solution method for detecting the presence of coding regions in DNA sequence and established superiority of the later technique [25-28]. The algorithm has been successfully tested on several sample databases downloaded from NCBI GenBank [29].

## 2 Materials and methods

PSD estimation of DNA sequence requires conversion of DNA character string into numerical form. Different researchers have adopted different mapping methods to achieve this objective. The Voss representation is a very popular technique giving four binary indicator sequences x_a_[n], x_t_[n], x_c_[n] and x_g_[n] which takes a value of either 1 or 0 at location n depending on whether the corresponding character exists at that location or not [7,13,14]. These indicator sequences show redundancy because

(1) $$x_{a}\left[n\right]+x_{t}\left[n\right]+x_{c}\left[n\right]+x_{g}\left[n\right]=1\;\mathrm{for}\;\mathrm{all}\;n$$

Therefore, three out of these four binary sequences would be enough to uniquely determine the DNA character string. There are several other techniques such as complex numbers [2], paired numeric [6], universal graphical representation [11], weak-strong hydrogen bonding [18], EIIP [30], quaternion [31] etc. each having a certain special feature of its kind. Rao and Shepherd [19] in their study found that complex mapping was one of the most effective and compact mapping rules. In a recent work, Kwan et al. [16] introduced several novel codes for single-sequence numerical representations for spectral analysis and studied their relative performances. They focused on direct and simple numerical representations which satisfied the following requirements:

(a). Single-sequence mapping for a nucleotide sequence

(b). Fixed value mapping for each nucleotide

(c). Accessible to digital signal processing analysis

Seven single-sequence complex-value numerical representations were derived by them in which each nucleotide of sequence was mapped to a single real value element (+1 or -1) and a single imaginary value element (+j or - j). According to the main findings of their study, the K-Quaternary Code-I was most attractive whereas Rao and Shepherd found K-Quaternary Code-III to be more suitable. Details of these codes are furnished in Table 1. In this article, the authors have adopted a novel mapping rule in which K-Quaternary Code-III has been flipped about *Y*-axis assigning numerical values, a = -1, c = -j, g = 1 and t = j to nucleotide sequence x[n] as shown in the following example in order to provide location accuracy to predicted exons.

(2) $$x\left[n\right]=\left[a\;t\;g\;c\;c\;t\;t\;a\;g\;g\;a\;t\right]$$

**Table 1:** Numerical representations

**Name**	**c**	**g**	**a**	**t**	**Remarks**
K-Quaternary Code-III	-j	-1	+1	+j	Rao and Shepherd
K-Quaternary Code-I	-1	-j	+1	+j	Kwan et al.
Quaternary Code proposed	-j	+1	-1	+j	Proposed mapping

After mapping,

(3) $$x_{m}\left[n\right]=\left[‒1\;j\;1‒j‒j\;j\;j‒1\;1\;1‒1\;j\right]$$

Once numerical conversion of DNA sequence is obtained, DSP technique can easily be applied to estimate its power spectrum. Spectral estimation by non-parametric method can be broadly classified as direct and indirect. These two methods are equivalent and are popularly known as the periodogram method. The direct method takes discrete Fourier transform (DFT) of the signal and then averages the square of its magnitude. The indirect method is based on the concept of first estimating the autocorrelation of data sequence and then taking its Fourier transform (FT).

In the first part of this section, spectral analysis of DNA by periodogram method is discussed in brief. The basic of eigendecomposition is given in the second subsection. Mathematical background of the least-norm solution is explained in the third subsection followed by algorithm of the least-norm solution technique. In the next section of this article, results and discussion have been presented. In the first subsection of this section, performance of proposed method has been compared with the modified periodogram method. Model order selection by eigenvalue ratio technique has been elaborated in the next subsection. In the final and last section of the article, conclusion has been drawn. MATLAB 7.1 software has been used to show performance of the estimators.

### 2.1 Spectral analysis by modified periodogram method

In the direct method mentioned above, periodogram *P*_per_(*f*_*k*_) for signal *x*(*n*) can be computed by DFT or more efficiently by fast Fourier transform (FFT) for *N* data points as shown in Equation 4:

(4) $$P_{\mathrm{per}}\left(k/N\right)=1/N|\sum_{n=0}^{N-1}x\left(n\right)e^{-j2\mathrm{\Pi nk}/N}|{}^{2}$$

$$\mathrm{where}\;f_{k}=k/N,\;\mathrm{for}\;k=0,1,2,\ldots ,N-1$$

To enhance performance of the periodogram method, at first, the *N*-point data sequence is divided into *K* overlapping segments of length *M* each, then the periodogram is computed applying the Bartlett window; finally, the average is computed from the result.

### 2.2 Spectral analysis by eigendecomposition

In this article, eigendecomposition of the autocorrelation matrix has been motivated as an approach for frequency estimation of DNA sequence. Here, the signal *x*(*n*) is modeled as a sum of *p* complex exponentials in white noise *w*(*n*) as shown in the following equation:

(5) $$x\left(n\right)=\sum_{i=1}^{p}A_{i}e^{\mathrm{jn}w_{i}}+w\left(n\right),$$

where amplitude *A*_*i*_ are complex values given by *A*_*i*_ = |*A*_*i*_| *e*^*jφ*^_*i*_ with *φ*_*i*_ being uncorrelated random variables that are uniformly distributed over the interval [*π*, -*π*]. The power spectrum of *x*(*n*) consists of a set of *p* impulses of amplitude |*A*_*i*_| at frequencies *w*_*i*_ for *i* = 1,2,3,…,*p* plus power spectrum of white noise *w*(*n*) having variance *σ*_*n*_^2^.

The *M* × *M* autocorrelation sequence of the process with lag size *M* is given by

(6) $$R_{\mathrm{xx}}\left(k\right)=\sum_{i=1}^{p}P_{i}e^{\mathrm{jk}w_{i}}+\sigma_{n}^{2}\delta \left(k\right),$$

where *P*_*i*_ = |*A*_i_|^2^ is the power in the *i*th component. Therefore, the autocorrelation matrix R_xx_ is the sum of autocorrelation matrix due to signal R_s_ and autocorrelation matrix due to noise R_n_ which may be written concisely as

(7) $$R_{\mathrm{xx}}=R_{s}+R_{n}={\mathrm{EPE}}^{H}+{\sigma_{n}}^{2}I,$$

where E = [e_1_, e_2_,…, e_p_] is an *M* × *p* matrix containing *p* signal vectors e_i_ and E^H^ signifies its Hermitian transpose. P = {P_1_, P_2,_…, P_*p*_} is a diagonal matrix of signal powers. The eigenvalues of R_xx_ is *λ*_*i*_ = *λ*_*i*_^*s*^ + *σ*_*n*_^2^ where *λ*_*i*_^*s*^ are eigenvalues of R_s_ having rank *p* corresponding to signal subspace and the last (M-p) eigenvalues approximately equal to *σ*_*n*_^2^ are noise eigenvalues. Hence, the eigenvalues and eigenvectors of R_xx_ may be divided into two groups as shown below. Assuming that the eigenvectors have been normalized to have unit norm, we may use spectral theorem to denote R_xx_ as

(8) $$R_{\mathrm{xx}}=\sum_{i=1}^{p}\lambda_{i}v_{i}{v_{i}}^{H}+\sum_{i=p+1}^{M}\lambda_{i}v_{i}{v_{i}}^{H}$$

**Figure 1:**
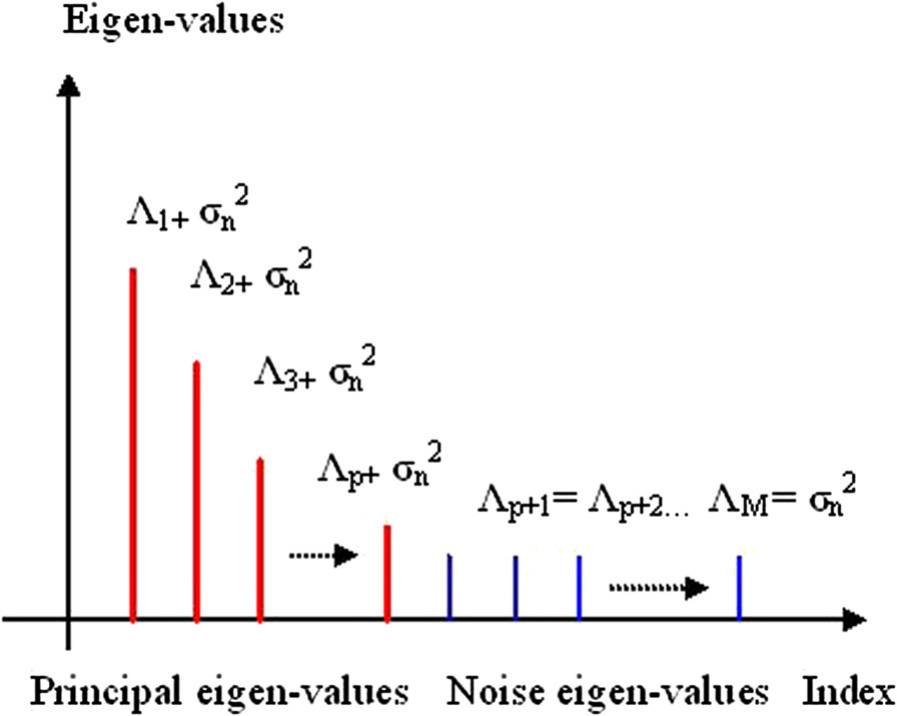
Decomposition of the eigenvalues of noisy signal into the principal and noise eigenvalues.

The set of eigenvectors {*v*_1_, *v*_2,_…, *v*_*p*_}, associated with largest eigenvalues span the signal subspace and are called principal eigenvectors. The second subset of eigenvectors {*v*_*p*+1_, *v*_*p*+2_,…, *v*_*M*_} span the noise subspace and have *σ*_*n*_^2^ as their eigenvalue. Since the signal and noise eigenvectors are orthogonal, it follows that the signal subspace and the noise subspace are also orthogonal. After eigendecomposition of the autocorrelation matrix, the eigenvalues are arranged in decreasing order *λ*_1_ ≥ *λ*_2_ ≥ *λ*_3_,…, ≥ *λ*_M_ as depicted in Figure 1. From this plot of eigenvalues, one can distinguish initial steep slope representing signal and a more or less flat floor representing noise level.

An issue that is of central importance to successful implementation of principal-component analysis (PCA) is the selection of appropriate model order *p* since the accuracy of estimated spectrum is critically dependent on this choice. In this article, the eigenvalue-ratio technique has been adopted for optimum model order selection. A plot of *λ*_*p*_/*λ*_*p*+1_ vs integer values *p* indicates a large eigenvalue gap at the threshold of signal subspace and noise subspace. This *p* value is chosen as the required model order and eigenvalues *λ*_*p*+1_ to *λ*_M_ are assumed to be the noise eigenvalues corresponding to the noise subspace.

The pseudo-spectrum estimation by noise subspace method involves three generic steps:

1. Formation of autocorrelation matrix from data vector.

2. Derivation of noise subspace with the help of eigendecomposition.

3. Identification of signal components from noise subspace by frequency estimation function.

### 2.3 Frequency estimation by least-norm solution

Frequency estimation is the process in which complex frequency components of a signal are estimated in the existence of noise [32]. The least-norm algorithm developed in this paper uses a single vector $\vec{a}$ that is constrained to lie on the noise subspace and the complex exponential frequencies are estimated from the peaks of the frequency estimation function:

(9) $${\hat{P}}_{\mathrm{LN}}\left(e^{\mathrm{jw}}\right)=1/{\left|{\vec{e}}^{H}\vec{a}\right|}^{2},$$

where {$\vec{e}$} is an auxiliary vector given by

(10) $$\vec{e}\;=\left[1\;e^{\mathrm{jw}}\;e^{j2w}\;e^{j3w}\ldots \ldots .e^{j\left(N-1\right)w}\right]$$

with $\vec{a}$ constrained to lie in the noise subspace, if the autocorrelation function is known exactly, then ${\left|{\vec{e}}^{H}\vec{a}\right|}^{2}$ will have nulls at the frequencies of each complex exponentials. Therefore, Z-transform of coefficients of $\vec{a}$ may be factored as

(11) $$A\left(z\right)=\sum_{k=0}^{M-1}a\left(k\right)z^{-k}\;=\prod_{k=1}^{p}(1-e^{\mathrm{jwk}}z^{-1})\prod_{k=p+1}^{M-1}(1-z_{k}z^{-1})$$

where Z_k_ for k = (p + 1),…,(M - 1) are the spurious roots that in general do not lie on the unit circle. The least-norm method attempts to eliminate the effects of spurious zeros by pushing them inside the unit circle leaving the desired zeros on the unit circle. The problem then is to determine which vector in the noise subspace minimizes the effects of spurious zeros on the peaks of ${\hat{P}}_{\mathrm{LN}}\left(e^{\mathrm{jw}}\right)$.

The approach used in the least-norm algorithm is to find a vector $\vec{a}$ that satisfies the three following constraints:

1. The vector $\vec{a}$ lies on the noise subspace ensuring that *p* roots of *A*(*z*) are on the unit circle.

2. The vector $\vec{a}$ has least Euclidean norm ensuring that spurious roots of *A*(*z*) lie inside unit circle.

3. The first element of $\vec{a}$ is unity, i.e. least-norm solution is not the zero vector.

**Figure 2:**
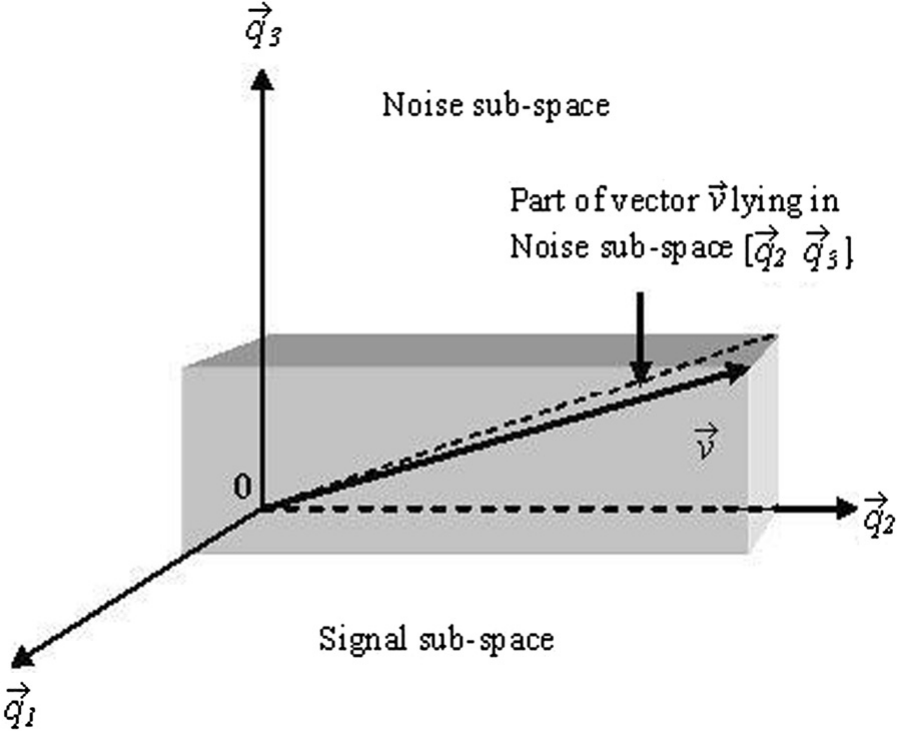
**Projection of signal vector**$\vec{v}$**on noise sub-space in a three -dimensional vector space.**

To solve this constrained minimization problem, we begin by noting the constraint that $\vec{a}$ lies on the noise subspace which is given by the following equation:

(12) $$\vec{a}=P_{n}\vec{v},$$

where $P_{n}=V_{n}V_{n}^{H}$ is the projection matrix projecting an arbitrary vector $\vec{v}$ on the noise subspace as shown in Figure 2[25].

The least-norm method involves projection of signal vector $\vec{v}$ on to the entire noise space.

The third constraint is expressed as

(13) $${\vec{a}}^{H}{\vec{u}}_{1}=1,$$

where

$${\vec{u}}_{1}={\left[1,0,0,\ldots ,0\right]}^{T}$$

This may be combined with the constraint in Equation 12 giving

(14) $${\vec{v}}^{H}\left({P_{n}}^{H}{\vec{u}}_{1}\right)=1$$

The norm of $\vec{a}$ may be written as

(15) $$||\vec{a}||{}^{2}=||P_{n}\vec{v}||{}^{2}={\vec{v}}^{H}\left(P_{n}^{H}P_{n}\right)\vec{v}$$

Since projection matrix *P*_*n*_ is Hermitian, therefore *P*_*n*_ = *P*_*n*_^H^ and also idempotent, hence *P*_*n*_^2^ = *P*_*n*,_ we get

(16) $$||\vec{a}||{}^{2}=||P_{n}\vec{v}||{}^{2}={\vec{v}}^{H}\left(P_{n}^{H}P_{n}\right)\vec{v}$$

Minimizing $\vec{a}$ is equivalent to finding vector $\vec{v}$ that minimizes the quadratic form of ${\vec{v}}^{H}P_{n}\vec{v}$

After reformulating the constrained minimization problem,

(17) $$i.e.,\;\min{\vec{v}}^{H}P_{n}\vec{v}\;\mathrm{subject}\;\mathrm{to}{\vec{v}}^{H}\left({P_{n}}^{H}{\vec{u}}_{1}\right)=1$$

Once the solution of Equation 14 is found, the least-norm solution is formed by projecting $\vec{v}$ onto noise subspace using Equation 12 and using Optimization Theory, the least-norm solution is found to be

(18) $$\vec{a}=P_{n}\vec{v}=\lambda P_{n}{\vec{u}}_{1}=\left(P_{n}{\vec{u}}_{1}\right)/\;{{\vec{u}}_{1}}^{H}P_{n}{\vec{u}}_{1}$$

which is the projection of the unit vector onto normalized noise subspace such that the first coefficient is unity, and the Lagrange multiplier *λ* is given by

(19) $$\lambda =1/\;\left({{\vec{u}}_{1}}^{H}P_{n}{\vec{u}}_{1}\right)$$

In terms of eigenvectors of the autocorrelation matrix, the least-norm solution is given using quadratic factorization (QR) by the following equation:

(20) $$\;\vec{a}\;=\left(\left(V_{n}V_{n}^{H}\right){\vec{u}}_{1}\right)/\;\left({{\vec{u}}_{1}}^{H}\left(V_{n}V_{n}^{H}\right){\vec{u}}_{1}\right)$$

### 2.4 Algorithm of proposed least-norm solution technique for estimating period-3 peaks

Step 1 Convert the samples of data vectors to column vector.

Step 2 Compute autocorrelation matrix of data with pre-determined lag size (M).

Step 3 Diagonalize the autocorrelation matrix. Produce diagonal matrix D of eigenvalues and a full matrix V whose columns are the corresponding eigenvectors so that X*V = V*D, where X is the signal matrix.

Step 4 Sort diagonal matrix D in ascending order for eigendecomposition. Take into account noise subspace spanned by the eigenvectors corresponding to nonsignificant eigenvalues.

Step 5 Project signal vector $\vec{v}$ onto the noise space using projection matrix.

Step 6 Find Least Norm vector $\vec{a}$ on noise subspace with first element equal to unity using QR factorization and applying the Optimization Theory.

Step 7 Estimate pseudo-spectrum (in dB) by computing absolute FFT of vector

$$\vec{a}$$

Step 8 Plot the result (in dB) to observe period-3 spectral peaks.

## 3 Results and discussion

The proposed algorithm has been tested on several eukaryotic genes to predict location of coding regions of varying lengths of a few base-pairs to thousand base-pairs and simulation results are compared with that of modified periodogram on the same DNA data. The segments of test data used for analysis contain both exons and introns of fully constructed genes. According to period-3 property of DNA, a prominent peak should be observed in the PSD plot of each exon segment. It is observed that the proposed method produces very sharp and well-defined period-3 peaks indicating existence and numbers of protein-coding regions of very short to long coding segments present in the test data. Once the existence and locations of exons in the enormous length of DNA are confirmed, further statistical or computational methods may be applied on the DNA sequence to find the boundaries of protein-coding regions. The statistical parameters and computation times for modified periodogram and least-norm methods for genes F56F11.4a, T12B5.1, C30C11 and D13156 are indicated in Table 2.

**Table 2:** Summary of statistical parameters and computation time of modified periodogram and least-norm methods for various genes

**Gene**	**Sliding DFT method**				**Least-norm method**			
	**Q.F.**	**CPU**	**Window**	***K***	**Q.F.**	**CPU**	**Model**	**Percent**
	**(mean)**^**2**^**/var**	**Time**	**Length**	**No. of**	**(mean)**^**2**^**/var**	**Time**	**Order**	**Rise in**
		**(s)**	***M***	**segments**		**(s)**	***p***	**Q.F.**
F56F11.4a	4.83	0.24	351	23	121.89	104.86	20	2.42e + 003
T12B5.1G-1	6.32	0.14	252	07	347.96	48.72	08	5.41e + 003
T12B5.1G-2	5.58	0.14	252	08	305.51	50.37	16	5.37e + 003
T12B5.1G-3	3.54	0.09	252	04	742.96	06.68	02	2.09e + 004
T12B5.1G-4	8.38	0.15	252	09	221.09	54.15	17	2.54e + 003
T12B5.1G-5	5.88	0.13	252	06	227.29	07.76	17	3.76e + 003
C30C11G-1	10.43	0.18	252	12	498.41	11.37	07	4.68e + 003
C30C11G-2	3.92	0.10	210	04	107.79	06.21	17	2.65e + 003
D13156	4.84	0.15	351	05	246.08	37.38	17	4.98e + 003

**Figure 3:**
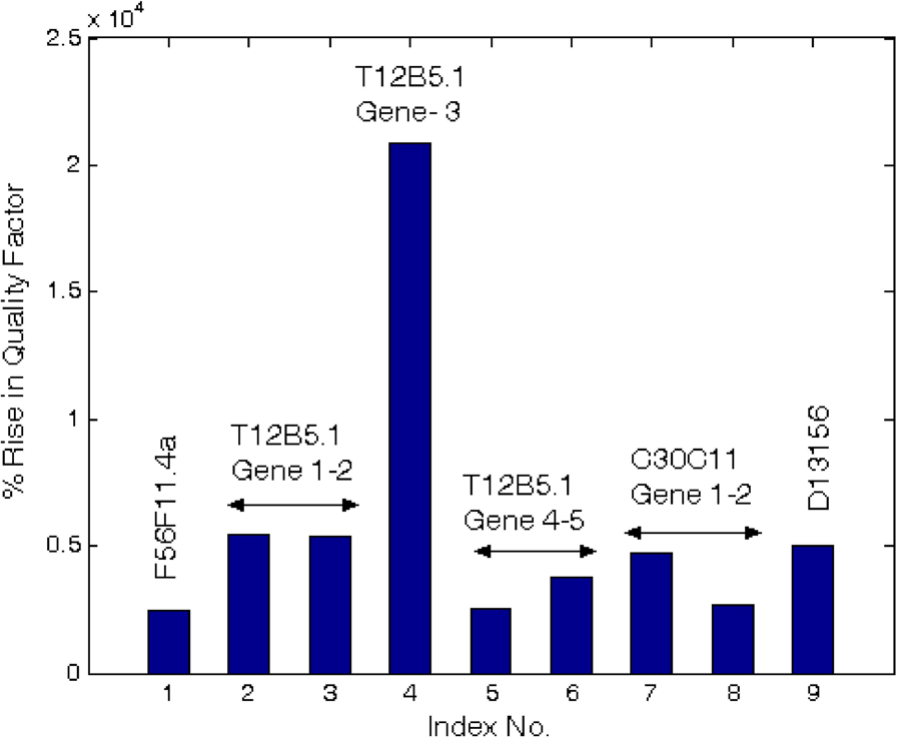
Percentage rise in quality factor for various genes by least-norm and periodogram methods.

It is observed that the proposed approach removes the entire noise and reveals the hidden periodicities prominently. A comparison has been drawn with periodogram method applying Bartlett (triangular) sliding window with 50% overlap and suitable segment lengths *M* and number of segments *K*. Window length *M* should be chosen subjectively based on a trade-off between spectral resolution and statistical variance. If *M* is very small, important features may be smoothed out, while if *M* is very large, the behavior becomes more like unmodified periodogram with erratic variation. Hence, a compromise value is selected between range 1/25 < *M*/*N* < 1/3 where *N* is nucleotide sequence length. Quality factor (Q.F.) which measures the ratio of variance to square of mean of PSD has been used as comparison metric between the two methods which are shown in Table 2. It is observed that quality factor of spectrum by the least-norm method is much higher than modified periodogram method. Figure 3 shows bar plot of percentage rise in quality factor for various genes. Table 2 also indicates that computation time required in the least-norm method is more than modified periodogram method.

### 3.1 Performance comparison of proposed method with existing method

The analysis of performance of both the methods can be made by prediction measures such as sensitivity (*S*_n_), specificity (*S*_p_), miss rate (*M*_r_) and wrong rate (*W*_r_). Their definitions are stated below:

(21) $$S_{n}=T_{p}/\left(T_{p}+F_{n}\right)$$

(22) $$S_{p}=T_{p}/\left(T_{p}+F_{p}\right)$$

(23) $$M_{r}=M_{e}/A_{e}$$

(24) $$W_{r}=W_{e}/P_{e}$$

where *M*_e_ = missing exons, *A*_e_ = actual exons, *W*_e_ = wrong exons, *P*_e_ = predicted exons, *T*_p_ = true positive, *F*_p_ = false positive, and *F*_n_ = false negative. *T*_p_ corresponds to those genes that are accurately predicted by the algorithm and also exist in the GenBank annotation. *F*_p_ corresponds to the exon regions which are identified by the given algorithm but are not specified in the standard annotation. *F*_n_ is coding region that is present in the GenBank annotation but is not predicted as a coding segment by the algorithm. The average value of *S*_n_ and *S*_p_ gives the overall exon sensitivity and specificity. Table 3 summarizes the simulation results of the eight genes used as test data. It is evident from tabulated data that *S*_n_, *S*_p_ and the average of *S*_n_ and *S*_p_ of the proposed method are significantly higher than existing method in all the cases whereas the miss rate and wrong rate are much lower indicating superior performance of the proposed algorithm over the existing technique [33].

**Table 3:** Summary of performance analysis of data for least-norm and modified periodogram methods

**Gene**	**DSP**	**Threshold**	**Prediction measures**				
	**methods**	**value**	***S***_**n**_	***S***_**p**_	**(*S***_**n**_ **+** ***S***_**p**_**)/2**	***M***_**r**_	***W***_**r**_
F56F11.4a	Periodogram	1.75	0.4	1.0	0.70	0.6	0.0
	Periodogram	1.50	0.8	0.66	0.73	0.2	0.4
	Least-norm	*	1.0	1.00	1.00	0.0	0.00
T12B5 Gene-1	Periodogram	1.75	1.0	0.43	0.71	0.0	0.55
	Periodogram	1.50	1.0	0.33	0.66	0.0	0.66
	Least-norm	*	1.0	1.0	1.0	0.0	0.0
T12B5 Gene-2	Periodogram	1.75	1.0	0.6	0.8	0.0	0.4
	Periodogram	1.50	1.0	0.5	0.75	0.0	0.5
	Least-norm	*	1.0	1.0	1.0	0.0	0.0
T12B5 Gene-3	Periodogram	1.75	1.0	0.15	0.57	0.0	0.84
	Periodogram	1.50	1.0	0.12	0.56	0.0	0.87
	Least-norm	*	1.0	1.0	1.0	0.0	0.0
T12B5 Gene-4	Periodogram	1.75	0.5	0.4	0.45	0.5	0.6
	Periodogram	1.50	0.75	0.33	0.54	0.25	0.66
	Least-norm	*	1.0	1.0	1.0	0.0	0.0
T12B5 Gene-5	Periodogram	1.75	0.66	0.22	0.44	0.33	0.77
	Periodogram	1.50	1.0	0.25	0.62	0.0	0.75
	Least-norm	*	1.0	1.00	1.00	0.0	0.0
C30C11 Gene-1	Periodogram	1.75	0.5	0.4	0.45	0.5	0.6
	Periodogram	1.50	1.0	0.4	0.7	0.0	0.6
	Least-norm	*	1.0	1.0	1.0	0.0	0.0
C30C11 Gene-2	Periodogram	1.75	1.0	0.33	0.66	0.0	0.66
	Periodogram	1.50	1.0	0.21	0.60	0.0	0.78
	Least-norm	*	1.0	1.0	1.0	0.0	0.0
D13156	Periodogram	1.75	1.0	0.22	0.61	0.0	0.77
	Periodogram	1.50	1.0	0.15	0.57	0.0	0.86
	Least-norm	*	1.0	0.5	0.75	0.0	0.5

*Threshold value not required.

**Figure 4:**
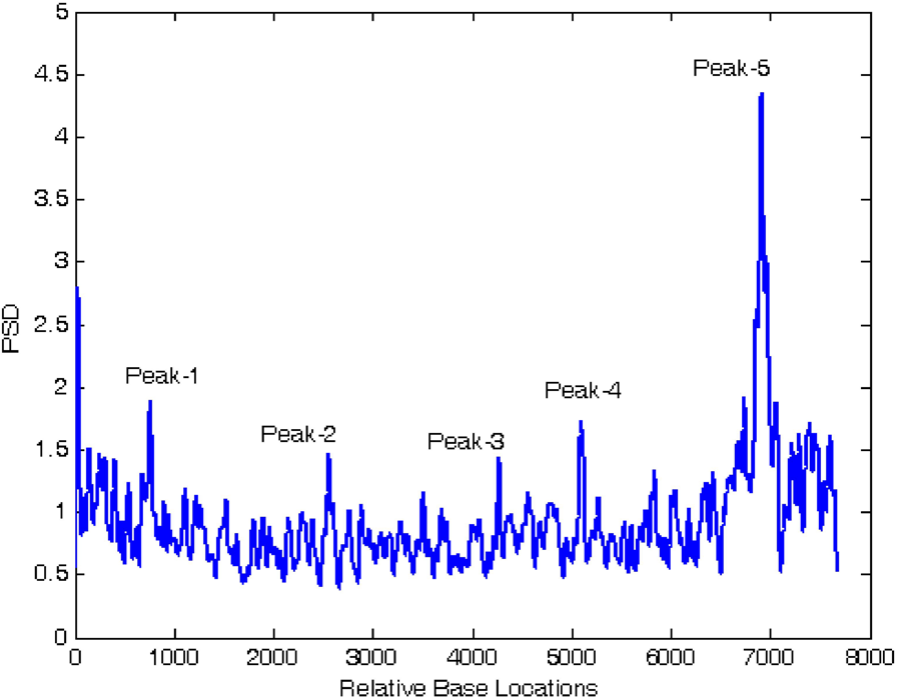
Plot of PSD by modified periodogram method for F56F11.4a gene.

**Figure 5:**
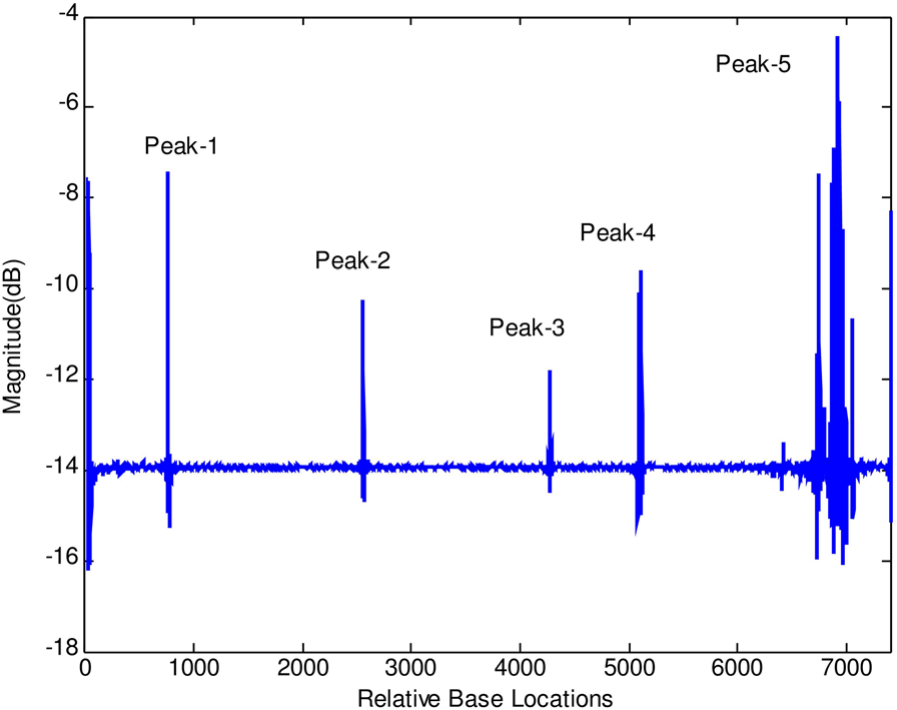
Plot of period-3 peaks by least-norm solution for F56F11.4a gene.

At first, both modified periodogram technique and proposed least-norm algorithm are applied to *C. elegans* cosmid F56F11.4a gene having 8060-base pair (bp) length test data starting from 7021-bp location. It has five known exons between locations 7948 to 8059, 9548 to 9877, 11,134 to 11397, 12485 to 12664 and 14275 to 14625 bp. The modified periodogram result is shown in Figure 4 and the proposed algorithm result is plotted in Figure 5. In the PSD plot shown in Figure 4, there are five visible exon peaks in the presence of background noise. But it is evident from Figure 5 by the proposed method that the five sharp period-3 spectral peaks visible in the specific coding regions are well defined, accurately positioned and without any noise component.

**Figure 6:**
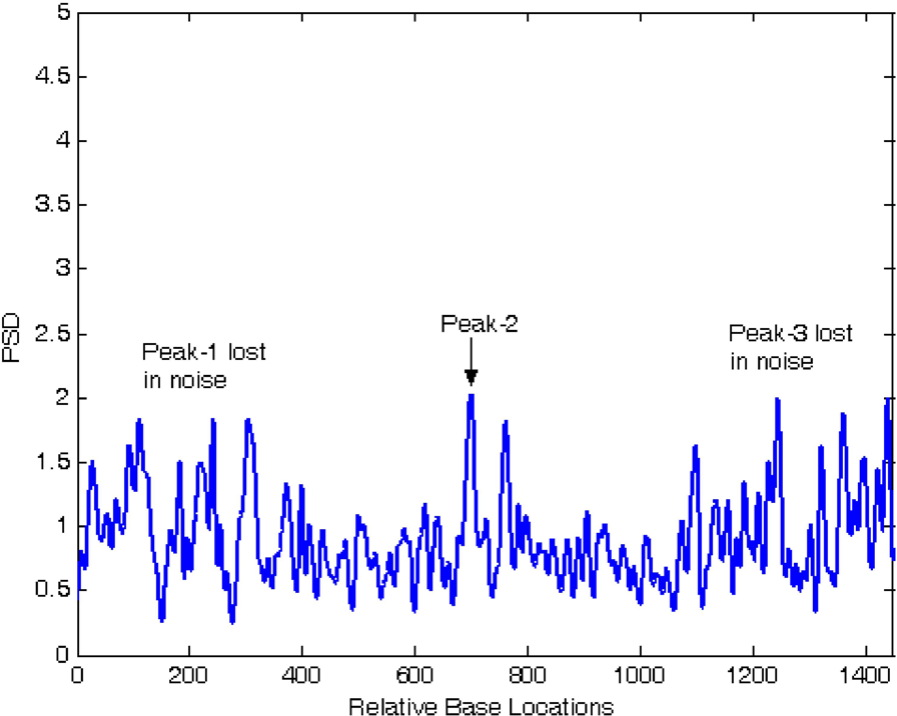
Plot of PSD by modified periodogram method for T12B5.1 gene-1.

**Figure 7:**
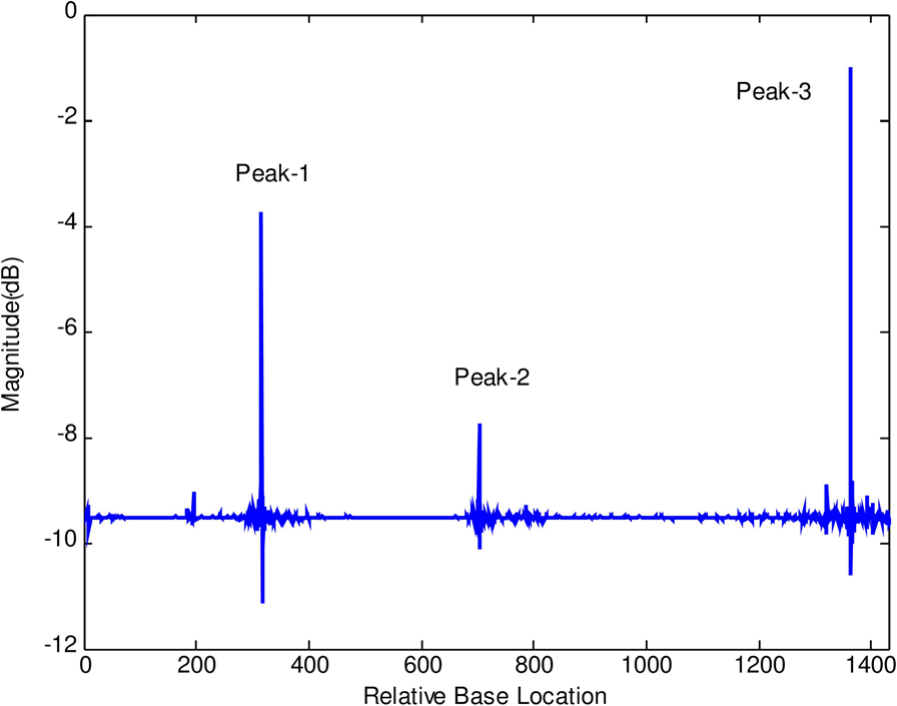
Plot of period-3 peaks by least-norm solution for T12B5.1 gene-1.

**Figure 8:**
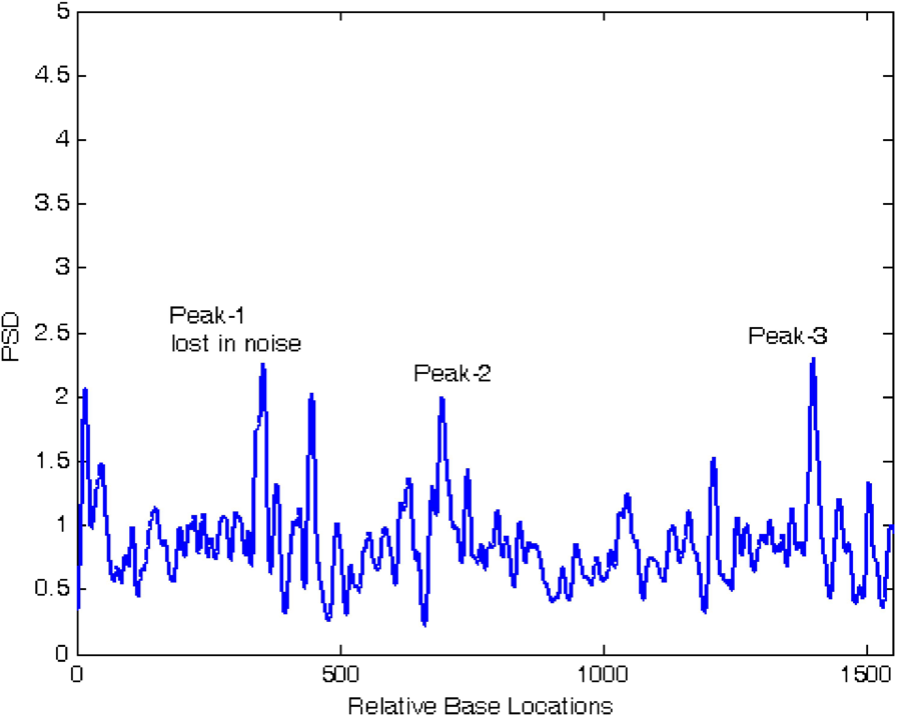
Plot of PSD by modified periodogram method for T125B.1 gene-2.

**Figure 9:**
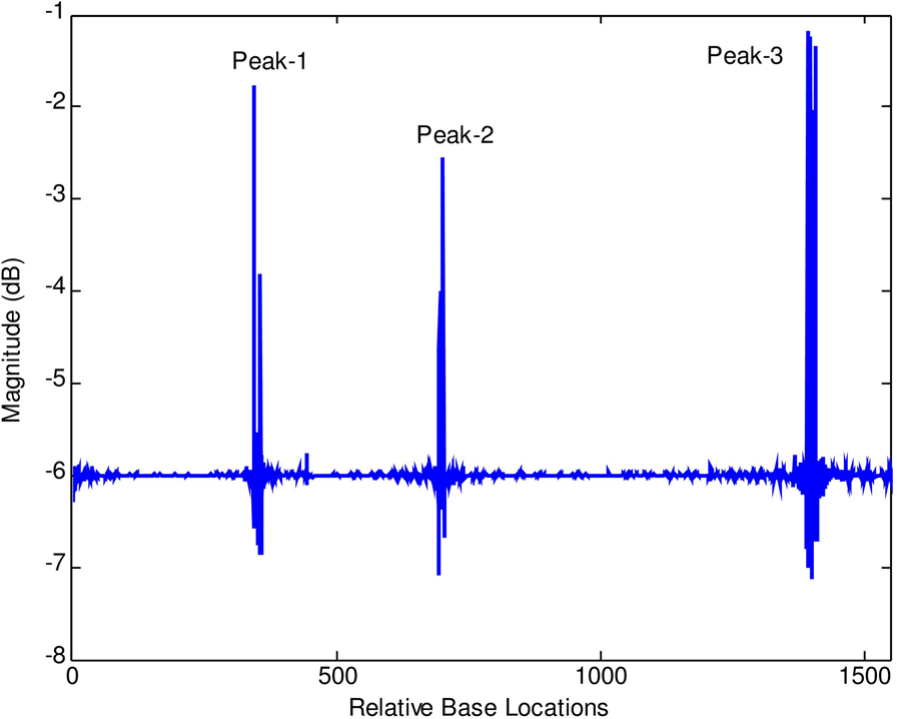
Plot of period-3 peaks by least-norm solution for T12B5.1 gene-2.

Figures 6 and 7 show the results of application of conventional modified periodogram method and proposed least-norm solution method to 32488-bp length *C. elegans* cosmid T12B5.1 DNA (Accession no. FO081674.1 AF100307). The plots indicate three exons in gene-1 between locations 17332 to 17402, 17645 to 18266, and 18311 to 18505 bp. In Figure 6, the exon peaks are present along with other peaks; therefore, prediction becomes ambiguous. In Figure 7, obtained by the proposed algorithm, there are only three sharp period-3 peaks corresponding to the exons present in the gene. They are in proper location and are absolutely devoid of noise. Hence, there is no scope of any ambiguity. Similar results are seen in Figures 8 and 9 for gene-2 with three exons between locations 18994 to 19064, 19349 to 19997 and 20059 to 20253 bp. The technique was applied to the remaining three genes of this DNA and was verified successfully.

**Figure 10:**
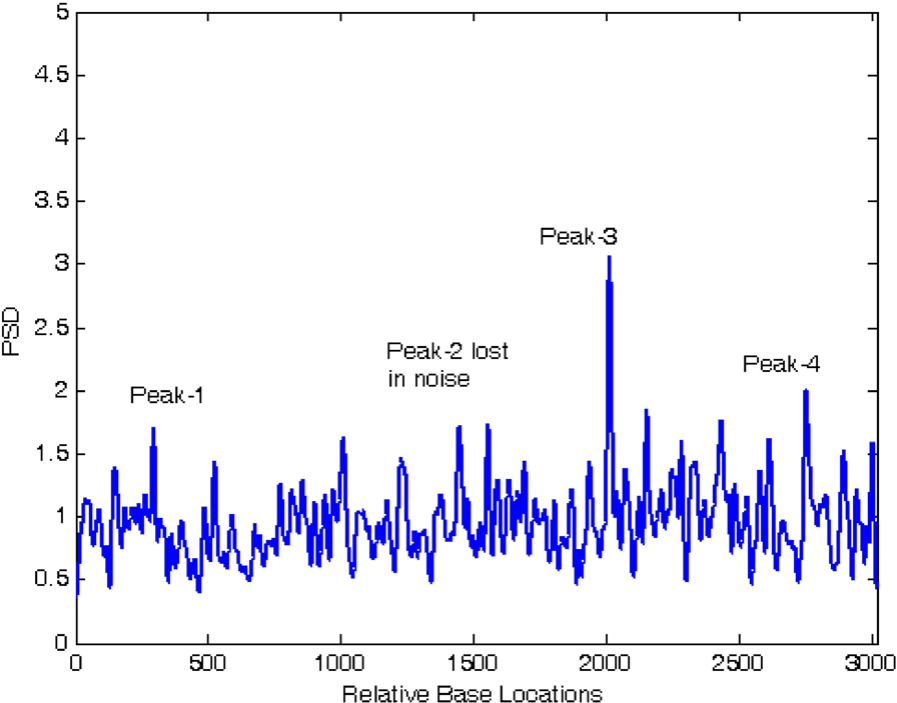
**Plot of PSD by modified periodogram method for *C. elegans *** **C30C11 gene-1.**

**Figure 11:**
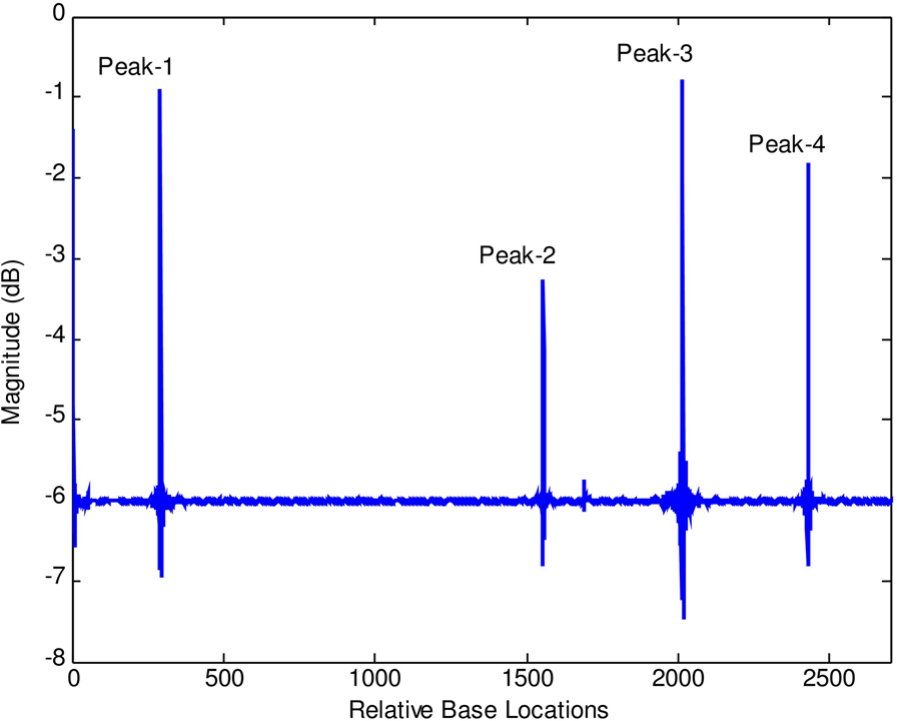
Plot of period-3 peaks by least-norm solution for C30C11 gene-1.

**Figure 12:**
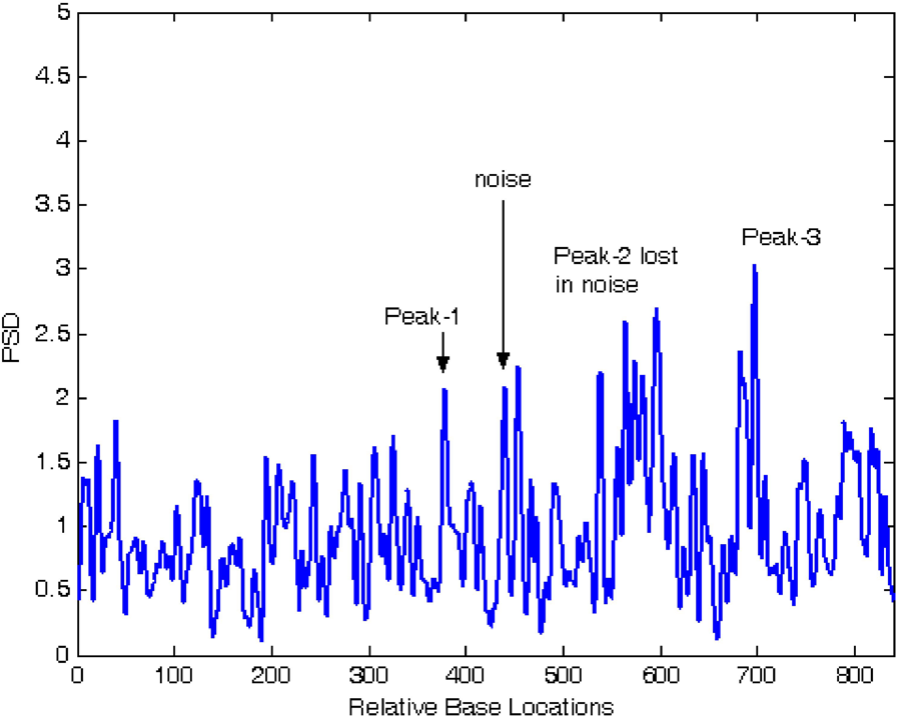
Plot of PSD by modified periodogram method for C30C11 gene-2.

**Figure 13:**
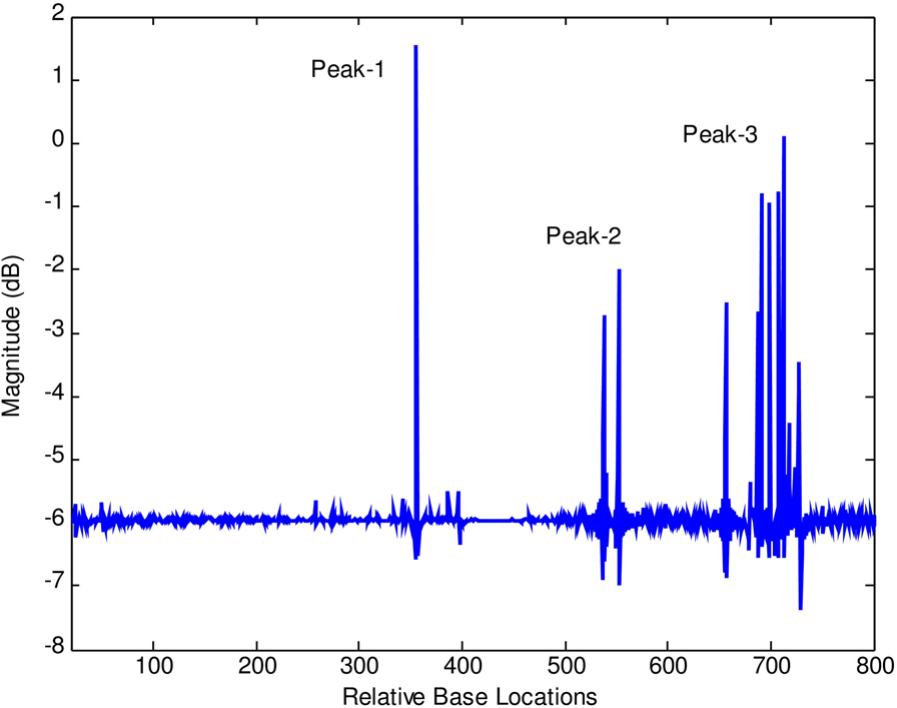
Plot of period-3 peaks by least-norm solution for C30C11 gene-2.

Next, both the methods were applied to DNA C30C11 (Accession no. FO080722.7 L09634) from *C. elegans*chromosome-III having length 30866 bp. Figures 10 and 11 mention spectral peaks by modified periodogram and least-norm solution method respectively for gene-1 with exons between locations 4874 to 4985, 5034 to 5408, 5452 to 6179 and 6227 to 6526 bp. In Figure 11 it is observed that peak-2 is shifted to right from actual position. Figures 12 and 13 indicate accurate results for gene-2 with exon segments between locations 7320 to7503, 7555 to 7757 and 7804 to 7923 bp. All these plots showing results of both the existing and proposed methods reflect the superiority of proposed technique over the conventional method because the peaks obtained with proposed algorithm are sharp, well defined, unambiguous, and noise-free. The threshold values for performance analysis of modified periodogram method have been chosen judiciously as 1.75 and 1.5, respectively. Table 3 indicates a list of genes studied and analysis summary of modified periodogram and least-norm solution approaches. In all the above examples cited, the proposed method shows better result than the existing method giving a higher value of sensitivity, specificity and their average as well as lower value of miss rate and wrong rate.

**Figure 14:**
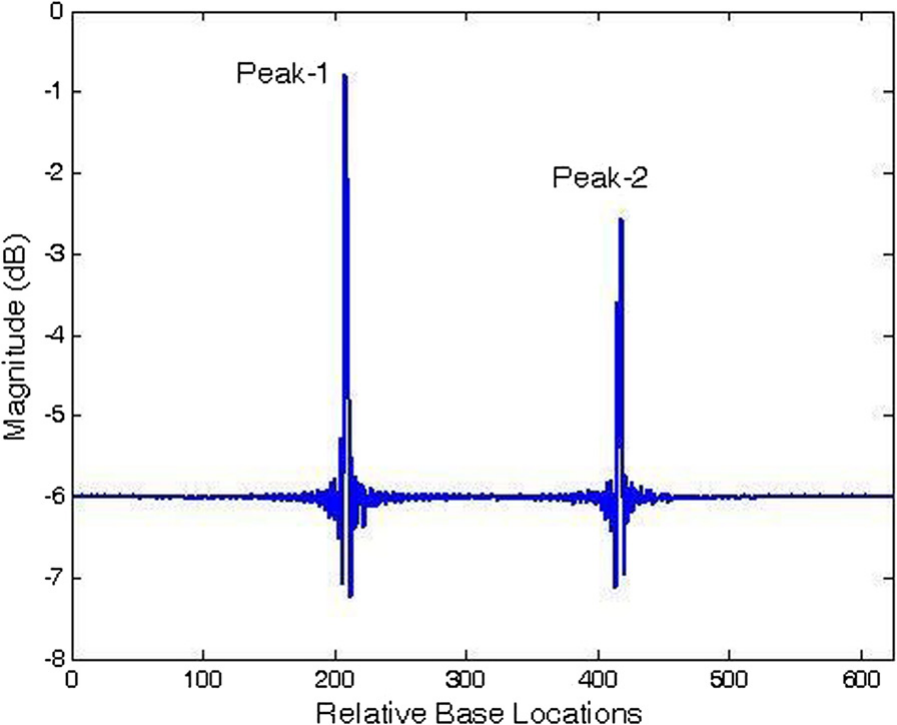
Plot of period-3 peaks by least-norm solution for DMPROTP1 gene.

**Figure 15:**
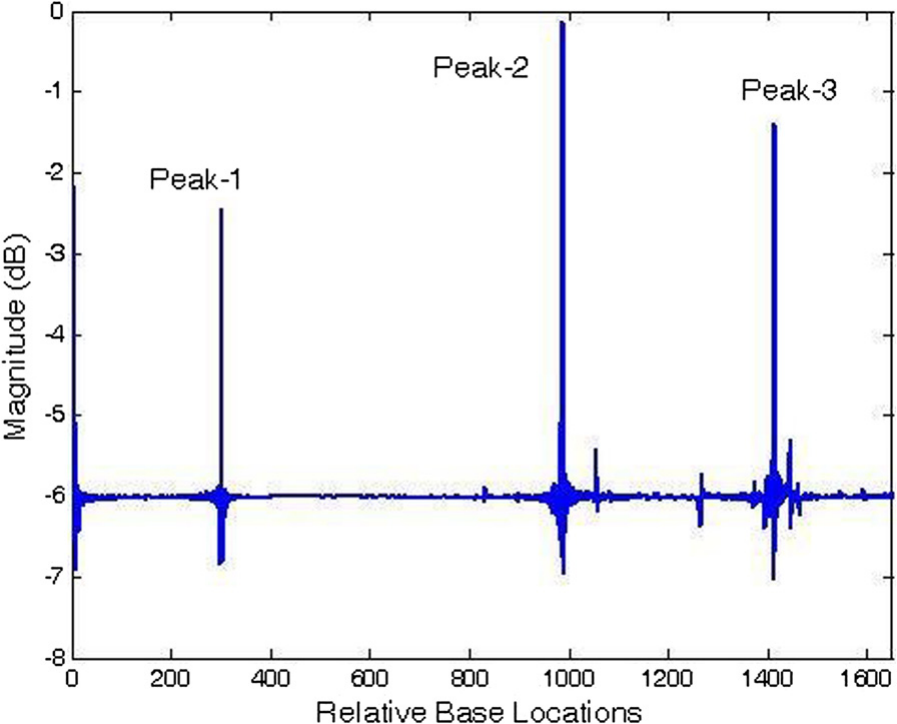
Plot of period-3 peaks by least-norm solution for CALEGLOBIM gene.

Next, least-norm algorithm has been applied to organisms with very short exon segments. It is known that prediction of exons with less than 100-bp length is difficult but the proposed least-norm method is found to be very suitable for detecting presence of exons as small as 28 bp length. Table 4 shows details of the organisms with short exons used as test data. Spectral plots for DMPROTP1 and CALEGLOBIM have been shown in Figures 14 and 15 respectively. The figures show very sharp, well defined and noise-free peaks in exon regions even for very small exon segments. Similar tests were performed on other organisms too giving satisfactory results. Hence, it is established that our method is robust and equally suitable for short as well as long exons.

**Table 4:** Details of organisms with short exons

**Gene ID**	**GenBank accession no.**	**DNA length in bp**	**Length of exons in bp**	**Source**
DMPROTP1	L17007.1	624	177 (122 to 248, 376 to 425)	*Didelphis marsupialis* (Southern opossum)
			Exon1-127 and Exon2-50	
OAMTTI	X07975.1	2055	186 (995 to 1022, 1312 to 1377, 1697 to 1,788) Exon1-28, Exon2-66, Exon3-92	*Ovis aries* (sheep)
CALEGLOBIM	L25363.1	1698	444 (144 to 235, 364 to 586, 1399 to 1527)	*Callithrix jacchus* (white tufted ear marmoset)
PIGAPAI	L00626.1	3333	Exon1-92, Exon2-223, Exon3-129	*Sus scorfa* (pig)
			798 (751 to 793, 975 to 1128, 1770 to 2,370)	
			Exon1-43, Exon2-154,	
			Exon3-601	

The proposed least-norm algorithm though offers high predictive accuracy compared to existing SDFT method, it has certain limitations on its part. It is a key issue to select model order judiciously for accurate exon detection. In the least-norm method, the time of execution is more compared to the other existing methods since computation time depends on the autocorrelation lag size which is determined depending on the length of nucleotide sequence being tested. The computation of many lags is required in estimation of periodicity which requires great deal of arithmetic, increasing the execution time of the proposed technique. It is desirable to exploit certain properties of autocorrelation function that are known to reduce the computational load. This can be done by taking advantage of the special technique based on reduction in number of multiplications given by Kendall [34]. Another method for speeding up the autocorrelation computation is by the well-known FFT method, which can also help in reducing computation time of proposed least-norm technique [35].

### 3.2 Eigenvalue-ratio based model order selection approach

**Figure 16:**
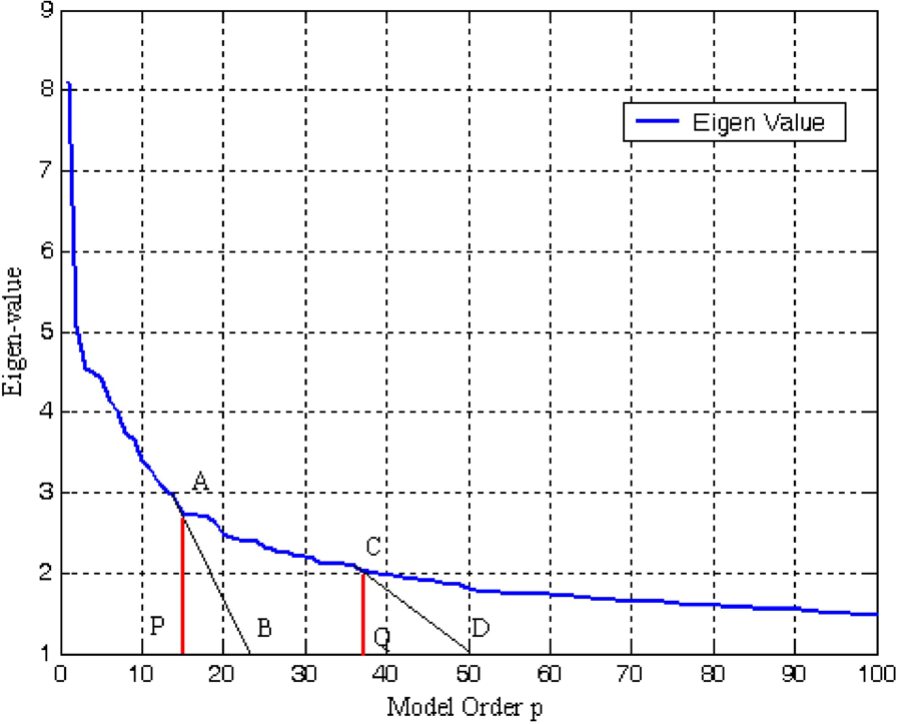
Plot of eigenvalue vs model order for F56F11.4a gene.

**Figure 17:**
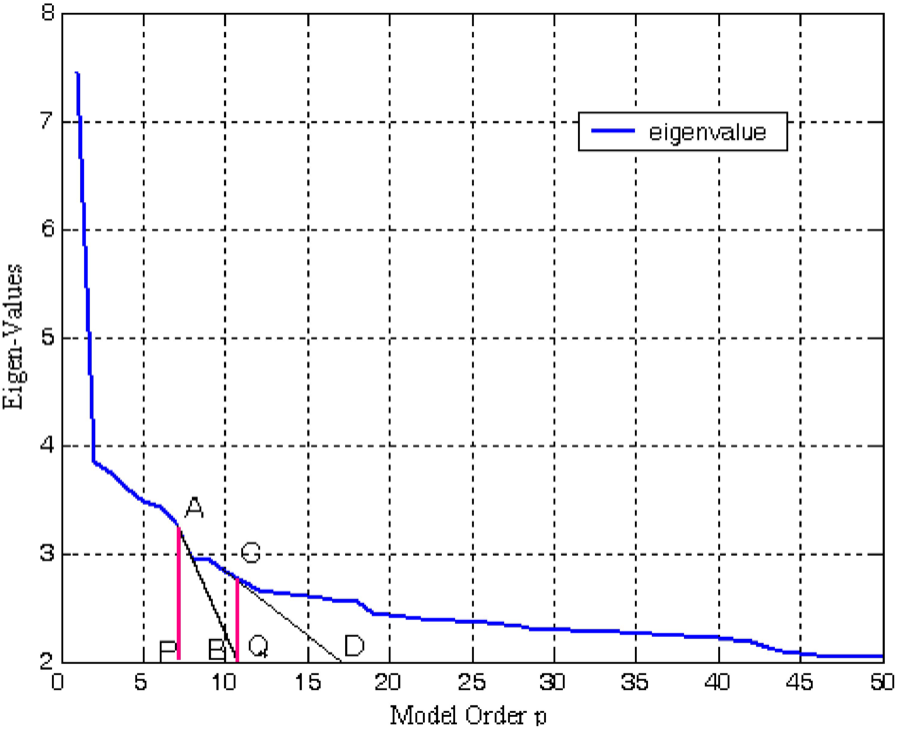
Plot of eigenvalue vs model order for C30C11 gene-1.

A key issue in developing the eigendecomposition-based model is proper selection of model order p. In order to estimate least-norm solution-based pseudo-spectrum, the dimension M-p of the noise subspace must be determined accurately. If value of p taken is less than required, then few prominent peaks may go unnoticed. On the other hand, if selected model order is more than the required value, undesired peaks are introduced in the plot leading to false prediction. The most common approach is to calculate and sort the eigenvalues of the correlation matrix R_xx_ of the noisy signal. The plot of eigenvalues sorted in decreasing order is termed as Scree-plot. The prime eigenvalues of dimension p having steep slope correspond to the signal subspace. The set of smallest eigenvalues having dimension M-p with values equal to noise variance *σ*_*n*_^2^ is more or less flat in nature (Figure 1). Decrease in negativity of the derivative from higher value to lower value is determined by the slope of tangents drawn from the Scree-plot to the *X*-axis. At first, two points are chosen carefully on the Scree-plot such that the first is on steep slope and second is on less steep portion of the eigen-curve. The values of model order *p* intercepted by the two projections drawn vertically downward from the point of the tangent touching the eigen-curve (Scree plot) to the *X*-axis are identified. A ‘large gap’ or ‘elbow’ is looked for within this segment by eigenvalue-ratio technique to be treated as the threshold value between signal and noise subspaces (Figures 16 and 17).

**Figure 18:**
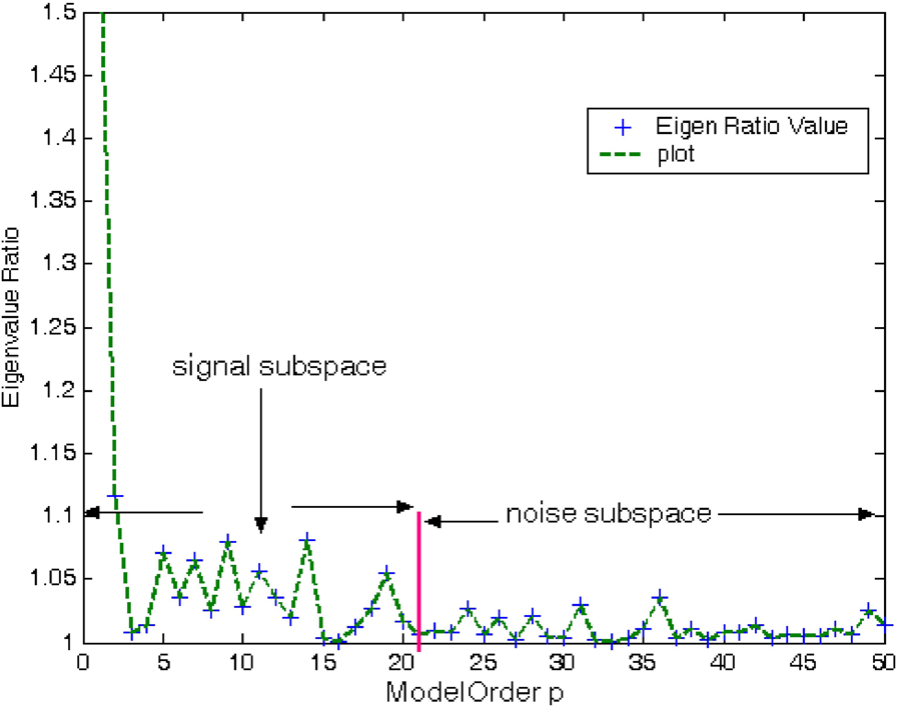
Plot of eigenvalue-ratio vs model order F56F11.4a gene.

**Figure 19:**
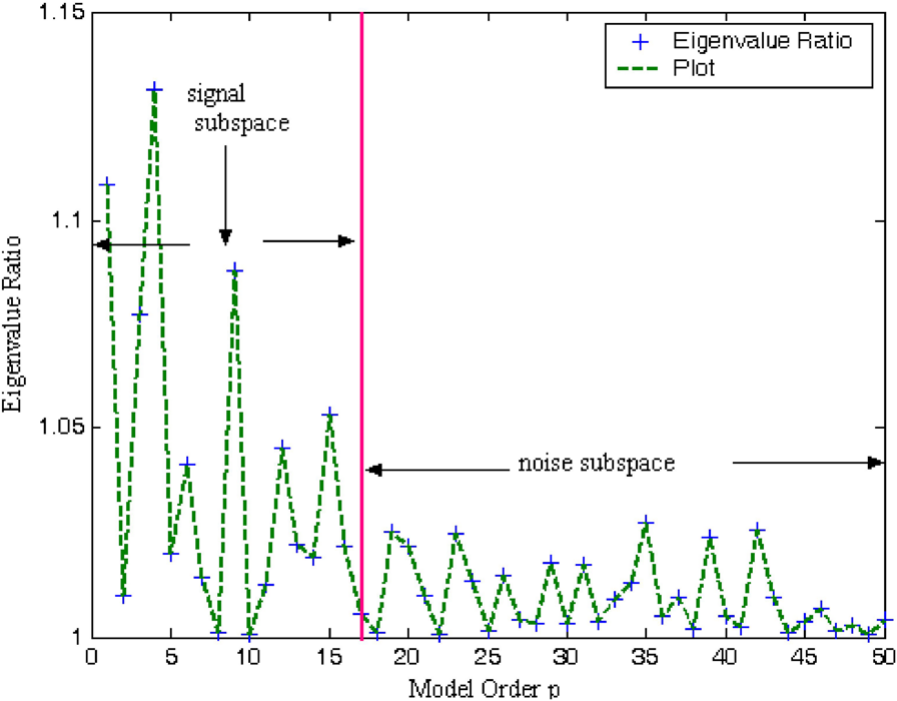
Plot of eigenvalue-ratio vs model order for T12B5.1 gene-2.

A very simple method based on eigenvalue ratio has been adopted by the authors to find model order p is discussed in this subsection [32,36]. As shown in Figures 18 and 19 the authors have plotted eigenvalue ratio *λ*_p_/*λ*_p+1_ vs model order p. It is noted that there exists an eigenvalue gap of high magnitude between orders *p* = 20 and 21 and *p* = 16 and 17, in the figures, respectively. Satisfactory estimates of rank of R_xx_ by suggested method was found to be 20 for F56F11.4a gene, 16 for T12B5.1 gene-2, and 7 for C30C11 gene-1 Thus, it may be considered that eigenvalues *λ*_21_, *λ*_17_ and *λ*_8_ onwards can be treated as noise eigenvalues in the three successive cases.

In this article, spectral content measure techniques based on sliding DFT was compared with proposed least-norm technique. In an early work, Tiwari et al*.* (1997) employed Fourier technique to analyze the three-base periodicity in order to recognize coding regions in genomic DNA. They observed that a few genes in *Saccharomyces cerevisiae* do not exhibit period-3 property at all. Anastassiou (2000, 2001) was inspired by the work of Tiwari et al. and introduced computational and visual tools for analysis of biomolecular sequences. He developed optimization procedure for improving performance of traditional Fourier technique. Later, Vaidyanathan and Yoon (2004) designed multistage narrowband band-pass filter for reducing background 1/f noise. Recently, Sahu and Panda (2011) in their work improved computational efficiency by employing SDFT with the help of the Goertzel algorithm, but the method is constrained by frequency resolution and spectral leakage effects.

The least-norm algorithm presented in this paper provides an absolutely novel approach. The first important feature of the proposed algorithm is that it produces very sharp and well-defined period-3 peaks in the protein-coding regions. The second significant feature is that it eliminates noise completely; hence, there is no requirement of setting threshold value. The third significant feature of this algorithm is that it is able to effectively detect very short exons as well. Moreover, this method offers very high sensitivity and specificity and very low miss rate and wrong rate compared to other available techniques.

## 4 Conclusion

DNA sequence analysis through power spectrum estimation by traditional non-parametric methods is in use since long. These are methodologically straightforward, computationally simple, and easy to understand, but due to low SNR, spectral features are difficult to distinguish as noise artifacts appear in spectral estimates. Therefore, effective identification of protein-coding region becomes difficult. The application of least-norm frequency estimator to capture period-3 peaks in coding regions has been introduced here. We used a constrained vector that lies on the noise subspace and the algorithm completely filters out the spurious peaks. Selection of proper model order is a fundamental issue in application of the eigendecomposition approach. The eigenvalue-ratio ‘gap’ or ‘elbow’ located on the Scree plot is treated as threshold between signal and noise spaces. Application of eigendecomposition-based methods to various DNA sequences has given amazing results as compared to standard classical methods in terms of resolution, quality factor, sensitivity, specificity, miss rate, and wrong rate. It was observed that high-resolution pseudo-spectrum estimator based on least-norm solution could identify protein-coding regions in DNA accurately. Another important feature of the proposed technique is that it can detect the presence of extremely short exon segments which is difficult for other existing methods. Unfortunately the computational effort for this high-resolution method is significantly higher than FFT processing. This limitation may be tackled by applying Kendall’s algorithm or incorporating the well-known FFT method to speed up the autocorrelation computation. Hence, it can be concluded that identification of protein-coding regions in DNA can be done effectively in a much superior way by applying the least-norm solution technique.

## Competing interests

The authors declare that they have no competing interests.
